# Time-Resolved Fluorescence Spectroscopy of Blood, Plasma and Albumin as a Potential Diagnostic Tool for Acute Inflammation in COVID-19 Pneumonia Patients

**DOI:** 10.3390/ijms241914703

**Published:** 2023-09-28

**Authors:** Tomasz Wybranowski, Blanka Ziomkowska, Michał Cyrankiewicz, Jerzy Pyskir, Maciej Bosek, Marta Napiórkowska, Marta Pilaczyńska-Cemel, Grzegorz Przybylski, Stefan Kruszewski

**Affiliations:** 1 Department of Biophysics, Faculty of Pharmacy, Collegium Medicum in Bydgoszcz, Nicolaus Copernicus University in Toruń, 85-067 Bydgoszcz, Poland; tomaszwybranowski@cm.umk.pl (T.W.); blanka@cm.umk.pl (B.Z.); mbosek@cm.umk.pl (M.B.); m.napiorkowska@cm.umk.pl (M.N.); skrusz@cm.umk.pl (S.K.); 2 Department of Lung Diseases, Neoplasms and Tuberculosis, Faculty of Medicine, Collegium Medicum in Bydgoszcz, Nicolaus Copernicus University in Toruń, 85-067 Bydgoszcz, Poland; mpilaczynska@wp.pl (M.P.-C.); gprzybylski27@gmail.com (G.P.)

**Keywords:** COVID-19, human albumin serum, fluorescence lifetime of blood, fluorescence lifetime of plasma, time-resolved fluorescence spectroscopy of blood, oxidative stress in COVID-19

## Abstract

Fluorescence lifetime measurements of blood or plasma offer valuable insights into the microenvironment and molecular interactions of fluorophores, particularly concerning albumin. Neutrophil- and hypoxia-induced oxidative stress in COVID-19 pneumonia patients leads to hyperinflammation, various oxidative modifications of blood proteins, and potential alterations in the fluorescence lifetime of tryptophan-containing proteins, especially albumin. The objective of this study was to investigate the efficacy of time-resolved fluorescence spectroscopy of blood and plasma as a prompt diagnostic tool for the early diagnosis and severity assessment of COVID-19-associated pneumonia. This study examined a cohort of sixty COVID-19 patients with respiratory symptoms. To investigate whether oxidative stress is the underlying cause of the change in fluorescence lifetime, human serum albumin was treated with chloramine T. The time-resolved spectrometer Life Spec II (Edinburgh Instruments Ltd., Livingston, UK), equipped with a sub-nanosecond pulsed 280 nm diode, was used to measure the fluorescence lifetime of blood and plasma. The findings revealed a significant reduction in the fluorescence lifetime of blood (diluted 200 times) and plasma (diluted 20 times) at 360 nm in COVID-19 pneumonia patients compared with their respective values recorded six months post-infection and those of healthy individuals. Significant negative correlations were observed between the mean fluorescence lifetime of blood and plasma at 360 nm and several severity biomarkers and advanced oxidation protein products, while a positive correlation was found with albumin and the albumin–globulin ratio. The time-resolved fluorescence spectroscopy method demonstrates the potential to be used as a preliminary screening technique for identifying patients who are at risk of developing severe complications. Furthermore, the small amount of blood required for the measurements has the potential to enable a rapid fingerstick blood test.

## 1. Introduction

There is still a need for non-invasive biomarkers that are rapid and can facilitate early diagnosis as well as the assessment of severity or mortality risk in COVID-19 patients. Autofluorescence spectroscopy has demonstrated potential as a tool for detecting and assessing various disease states through the analysis of blood components [1,2,3,4,5]. Despite its initial expectations, the use of autofluorescence spectroscopy for clinical diagnosis and monitoring with blood samples, without the need for exogenous probes or labels, remains in the early stages of development. The clinical applicability of fluorescence changes is limited due to an incomplete understanding of the underlying mechanisms. Further research is needed to determine the sensitivity, specificity, and reproducibility of this technique for different disease states. Most techniques employed by researchers and considered as potential clinical methods focus on analyzing both endogenous fluorophores in plasma or serum, and those extracted from red blood cells (RBCs) using acetone. Steady-state fluorescence spectroscopy is the most frequently implemented tool in these studies. However, the fluorescence of blood alone is not typically used as a diagnostic tool. The fluorescence intensity measurements of blood are difficult to apply for distinguishing pathological states due to the high turbidity of the samples, leading to high variability in light scattering and complicating accurate measurements. Time-resolved measurements can overcome this disadvantage and be applied to investigations of blood or plasma since the fluorescence lifetime does not directly depend on fluorophore concentration or brightness. This method involves the precise measurement of the time between the excitation pulse and photon emission using time-correlated single photon counting (TCSPC). The statistical distribution of these times for a single-component sample can be described as an exponential function. The fluorescence decay curve of the entire sample is analyzed by fitting a single-exponential function (for homogeneous samples) or a multi-exponential function (in the case of more complex systems) to extract the amplitude and lifetime of different types of fluorophores. Amplitude can be used to estimate the relative abundance of the different types of fluorophores. The lifetime of each component can provide information on the average duration of the corresponding fluorophore’s excited state [6,7,8].

Blood is a complex fluid that contains various cellular and non-cellular components. The cellular components include red blood cells (erythrocytes), white blood cells (leukocytes), and platelets (thrombocytes). The non-cellular components are present in plasma, which is the liquid part of blood. Plasma contains water, ions, and a diverse array of biochemical substances, including proteins, nucleic acids, lipids, and carbohydrates, that perform vital functions in the body. The fluorescence of blood and plasma upon excitation at 260–290 nm is mainly determined by tryptophan residues and, to a small extent, by tyrosine. The contribution of phenylalanine to the fluorescence is negligible due to its low absorptivity and poor fluorescence yield [9,10,11,12]. Tryptophan is an amino acid commonly found in proteins, including human serum albumin (HSA) and globulin, which are the most abundant proteins in human blood plasma. A study analyzing blood plasma protein fractions revealed that about 85% of the fluorescence originating from tryptophan is derived from HSA (70%) and immunoglobulin (15%) [12]. For example, in albumin, the only tryptophan residue is located in the physiologically important subdomain 2A ligand binding site in its inner hydrophobic region. The fluorescence lifetime of blood or plasma can vary between individuals depending on their physiological or pathological conditions since this parameter is sensitive to the local environment in which the fluorophore resides [13]. It is reasonable to hypothesize that oxidative damage to tryptophan-containing proteins, i.e., albumin, may alter the fluorescence lifetime of blood or plasma. Albumin contains a single free cysteine residue (Cys34), which can participate in redox reactions [14]. Under oxidative stress conditions, the thiol group of this cysteine can be oxidized, leading to the formation of various products like sulfenic acid (−SOH), sulfinic acid (−SO2H), and sulfonic acid (−SO3H). Additionally, the cysteine residue can form disulfide bonds, leading to alterations in the protein’s conformation. Carbonylation is another type of modification that can occur in albumin under oxidative stress conditions. Protein carbonylation is a process where carbonyl groups (−C=O) are introduced into a protein, typically through the oxidation of amino acids such as lysine [15]. Furthermore, the attachment of peroxidation products to albumin can influence the local environment of tryptophan residues, thereby potentially modifying their fluorescence properties. It has been shown that during the oxidative burst, activated neutrophils release myeloperoxidase (MPO), an enzyme that catalyzes the formation of reactive halogen species (RHS), including hypochlorous acid (HOCl), from hydrogen peroxide and halide ions. It leads to oxidative modifications of proteins, such as human serum albumin (HSA), by targeting and altering specific amino acid residues [16,17,18,19]. Intriguingly, HOCl-modified HSA has been demonstrated to stimulate neutrophils for NADPH oxidase activation, thereby producing more reactive oxygen species (ROS) [20]. Many studies have shown increased levels of activated neutrophils in peripheral blood, with excessive neutrophil extracellular traps (NETs) formation observed in COVID-19 patients in response to infection [21,22,23]. On the other hand, it has also been suggested that increased oxidative stress is involved in COVID-19 pathophysiology [21,24,25,26,27,28]. Our previous study demonstrated that there is an association between elevated levels of advanced oxidation protein products (AOPPs) and inflammation during the course of COVID-19-related pneumonia [29]. Thus, inflammation and oxidative stress are interrelated and mutually reinforce each other, creating a feedback cycle. This cycle can contribute to the “cytokine storm” and can lead to complications such as acute respiratory distress syndrome (ARDS) and multi-organ failure [21,28,30]. Furthermore, hypoxia-induced signaling pathways in COVID-19 can modulate oxidative stress and increase excessive reactive oxygen species (ROS) generation. As a result, inflammation is exacerbated, which further indirectly promotes oxidative damage of proteins including albumin in the blood [31,32]. In our previously published results, an inverse correlation was observed between the mean fluorescence lifetime of plasma at 350 nm and the levels of oxidative markers in healthy subjects (e.g., thiobarbituric acid-reactive substances, ischemia-modified albumin, carbonyl groups) [33]. Thus, we hypothesize that measuring the fluorescence lifetime of blood or plasma could potentially provide information about the degree of inflammation in the acute phase of infection and indicate the severity of the disease. This study aimed to determine if the fluorescence lifetime of blood and plasma at 360 nm is shortened in patients hospitalized with COVID-19-related pneumonia. The decay time of the fluorescence on admission was compared with values recorded for the same patients one week later, as long as they were in the acute phase of infection, and 6 months post-infection. Furthermore, our purpose was to assess correlations between the mean fluorescence lifetime of diluted blood or plasma and inflammatory biomarkers, radiological scores of high-resolution computed tomography (HRCT), biomarkers of multi-organ impairment, other surrogate markers of severity or mortality in COVID-19, including albumin, and AOPPs. To the best of our knowledge, there are no reports of using time-resolved fluorescence measurements of the whole, diluted blood in COVID-19 patients or patients with pneumonia in general. This study is a preliminary trial. Developing reliable and minimally invasive diagnostic tools for COVID-19 and pneumonia patients is of utmost importance, and fluorescence lifetime measurements may offer promising results for further research.

## 2. Results

### 2.1. Time-Resolved Fluorescence Spectroscopy Measurements

**Figure 1:**
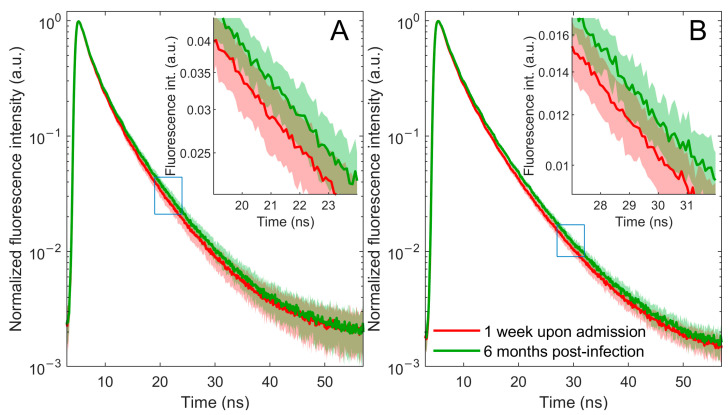
Fluorescence decay curves of blood (**A**) and plasma (**B**) samples from patients with COVID-19-related pneumonia 1 week upon admission (red line) and 6 months post-infection (green line). Averaged normalized decay curves with corresponding one-standard-deviation bands. The error bands are represented using a lighter shade of the appropriate color. A portion of the main figure is magnified and displayed as an inset for closer examination.

This study focuses on changes in fluorescence at 360 nm since both plasma and blood excited with a 280 nm light wavelength emit their most intense fluorescence in this area. The main objective of this research is to record and analyze the fluorescence decay curve, given its reduced sensitivity to factors such as sample turbidity that may affect fluorescence intensity. The data obtained from the TCSPC method, after zero-max normalization, revealed a faster fluorescence decay in patients with COVID-19-related pneumonia during the acute phase of infection, 1 week after admission, compared with the same patients’ data 6 months post-infection (Figure 1).

**Figure 2:**
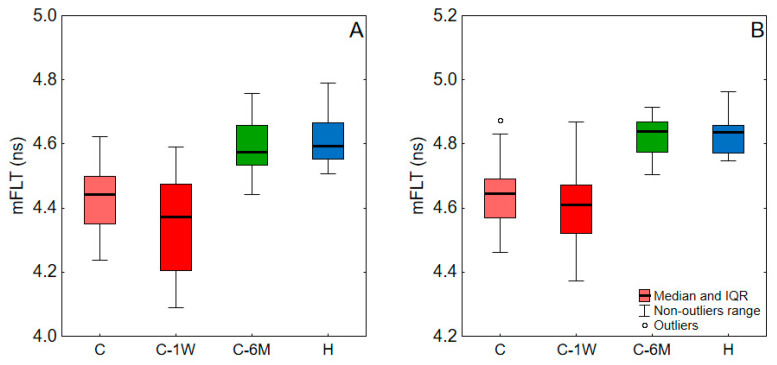
The mFLT of blood (**A**) and plasma (**B**) values for a study group. C, COVID-19 patients on admission to hospital; C-1W, COVID-19 patients 1 week upon admission; C-6M, COVID-19 patients 6 months post-infection; H, Healthy. Data outside the 1.5 interquartile range (IQR) from the first or third quartiles were considered outliers.

The mFLT of blood and plasma for COVID-19 patients at admission, one week upon admission, and six months post-infection, as well as for healthy subjects, are shown in Figure 2, and the levels of significance of the difference between these groups are shown in Table 1. There was a decrease in mFLT during the one-week hospital stay, which was significant only for blood. However, after six months, there was a notable increase in the mFLT of blood and plasma, significantly exceeding the levels observed during infection and reaching those in healthy individuals.

### 2.2. Clinical and Correlation Study

The clinical parameters typically used to predict mortality and severity in COVID-19 patients [34] are shown in Table 1. Most of these parameters for patients with COVID-19-related pneumonia in our study were outside their reference ranges. Table 2 also shows the correlation between these parameters and mFLT values. For both blood and plasma, mFLT was found to be negatively correlated with D-dimers, troponin, aspartate transaminase (AST), and AOPPs, while it was positively correlated with albumin and the albumin to globulin ratio (AGR). Moreover, in the blood samples, mFLT was negatively correlated with C-reactive protein (CRP), white blood cells (WBC), procalcitonin (PCT), lactate dehydrogenase (LDH), and the radiological score of computed tomography (HRCT). In the plasma samples, mFLT was also positively correlated with red blood cells (RBC).

### 2.3. In Vitro Study

**Figure 3:**
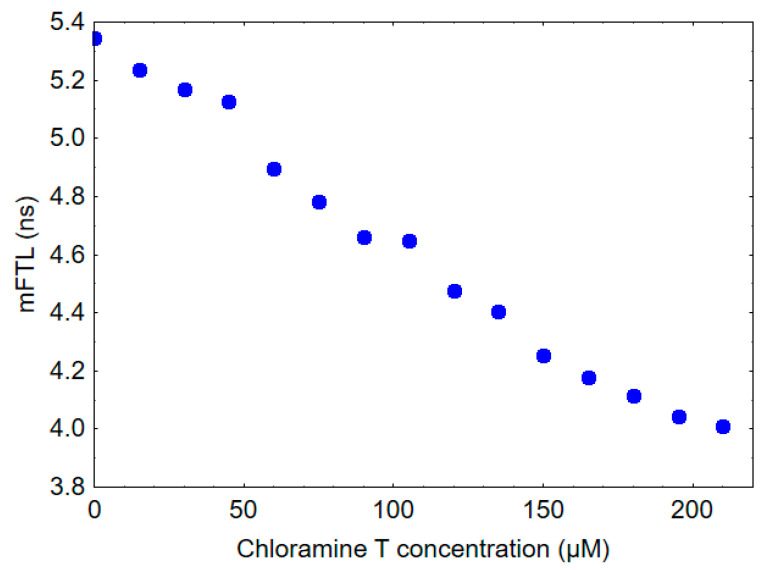
Effect of chloramine T exposure on mFLT of 10 µM HSA solution.

The mFLT of HSA was measured to be 5.3 ns, whereas the mFLT of human gamma globulin was found to be 2.7 ns. To provide some insights into the potential impact of oxidative stress on the fluorescence lifetime of blood and plasma, HSA was treated in vitro with increasing concentrations of chloramine T. As shown in Figure 3, the addition of chloramine T to HSA resulted in a dose-dependent decrease in mFLT.

## 3. Discussion

Tryptophan in albumin plays a major role in the fluorescence of blood and plasma at 360 nm after excitation at 280 nm. The literature indicates that the intrinsic fluorescence decay of tryptophan in water is bi-exponential as it involves two forms of excited-state tryptophan [35]. When tryptophan is in a protein environment, the fluorescence decay becomes more complex due to the interaction between the tryptophan residue and the surrounding protein matrix. As a result, the fluorescence decay of tryptophan in a protein environment can have multiple lifetimes and components, depending on the protein structure and composition. For instance, it has been reported that tryptophan’s fluorescence lifetime decay curve in HSA can consist of at least three components. The origin of tryptophan fluorescence lifetimes in proteins is a topic of ongoing debate among researchers. Several hypotheses have been proposed to explain the observed fluorescence lifetimes and their variations in different protein environments [36,37,38]. In the case of blood or plasma, individual fluorescence lifetimes obtained from the fitting analysis are not expected to reflect the individual sample components. Developing a more comprehensive model to accurately analyze fluorescence lifetime data in biological samples like blood or plasma can be challenging and requires more extensive experimental and computational approaches. The distribution of fluorescence lifetimes in such a system is highly complex, arising from the fact that the fluorescence of tryptophan originates from various proteins in the blood, each with different microenvironments. Furthermore, the fluorescence properties of tryptophan, such as its quantum yield, lifetime, and emission spectrum, can be influenced by changes in protein conformation. Fluorescent resonant energy transfer (FRET) between tryptophan and nearby tyrosine residues is possible and could introduce additional complexity in the decay pathways. Due to the impossibility of building an accurate model of plasma and blood fluorescence, and due to the numerical limitations of the applied decomposition procedure, further analysis was based on three- and four-exponential models. Incorporating further components into the model did not improve the quality of fit (chi-square value). Adding more components for the sake of capturing this complexity may not only risk overfitting but also render the computational process impractically intricate for the scope of this study. Since the subject of the study was global changes in plasma and blood fluorescence, based on the coefficients (optimized parameters) of the model, the mean fluorescence lifetime (mFLT) weighted by the fractional contribution of each component was calculated. We acknowledge that the use of mean fluorescence lifetime based on a three- or four-exponential fit might be an oversimplification of the complex fluorescence decay mechanisms in play. However, additional investigations into the multi-exponential model revealed that the incorporation of further decay components did not significantly alter the calculated mFLT value. This supports the assertion that our selected model is sufficiently comprehensive for capturing the essential fluorescence decay characteristics of the system, without unnecessarily overcomplicating it. The fluorescence lifetime obtained from this calculation could be considered an overall average or apparent lifetime. It should be noted that mFLT may not fully reflect the intricacies of the sample, but it can still provide valuable information about the fluorescence properties and behavior of the system. The approach taken here is intended to give a broad picture of the fluorescence characteristics.

Based on the significant correlations observed between the mFLT of blood and clinical parameters (Table 2) examined on admission, time-resolved fluorescence spectroscopy may potentially offer a rapid means of differentiating between mild and severe cases. It is important to note that in our study group, D-dimers exhibited the strongest negative correlation with the mFLT of the blood (r = −0.524). D-dimers are byproducts formed during the degradation of fibrin, which occurs as part of the blood clotting process. Elevated levels of D-dimers are associated with hypercoagulation complications and venous thrombotic events, which are widely recognized as major causes of mortality in individuals infected with SARS-CoV-2 [39,40]. Monitoring mFLT levels can be useful in identifying individuals who are at higher risk of developing these complications and may require more aggressive management, such as anticoagulant therapy.

In the course of our research, we examined the mFLT levels in both blood and plasma from individuals suffering from pneumonia associated with COVID-19 during the active phase of the infection. These levels were then compared with those observed long after recovery. This comparison led us to propose a close relationship between mFLT and the pathophysiology of COVID-19 infection, taking into account that the demographic characteristics and comorbidities of the participants in the study did not significantly change after six months. It is evident that some of the patients hospitalized due to pneumonia persistently experience chronic inflammation as a consequence of their underlying co-morbidities. Based on the fact that these patients, after recovering from COVID-19, had mFLT values similar to those registered in healthy individuals without any diseases, it can be concluded that only acute inflammation, not chronic inflammation, can affect changes in mFLT values.

The mechanism associated with the shortened mFLT of blood and plasma in COVID-19-related pneumonia patients is extremely complex and likely multifactorial, necessitating further research for a complete understanding. This phenomenon may be related to oxidative modifications of tryptophan-containing proteins, especially albumin. Our previous study on healthy subjects revealed that a decrease in mFLT at a wavelength of 350 nm could be associated with oxidative stress [33]. The decrease in the mFLT of HSA observed after chloramine T treatment, as shown in Figure 3, provides evidence supporting this hypothesis. Chloramine T is a known oxidizing agent, and its interaction with HSA in this experiment can be used to model oxidative stress conditions. The mFLT of HSA decreased in a dose-dependent manner following in vitro exposure to different concentrations of chloramine T. Activated neutrophils, important immune cells playing a key role in the inflammatory response to COVID-19, combat invading pathogens through the production of various reactive halogen species (RHS), including also chlorine compounds. The most significant chlorine compound produced by activated neutrophils is HOCl, formed by the reaction of hydrogen peroxide H2O2 with chloride ions (Cl-) in the presence of the enzyme myeloperoxidase (MPO) [17,41,42]. Chloramine T is often used in vitro for protein oxidation as an alternative to HOCl. It is a more stable compound that acts as a source of electrophilic chlorine, enabling selective oxidation of protein side chains. This property makes it a valuable tool for investigating the effects of oxidative stress on proteins, including HSA, under controlled experimental conditions. It is important to note that the in vitro experiment may not fully represent in vivo conditions; however, it provides a useful model to study the potential effects of oxidative stress on HSA and fluorescence lifetime decay of plasma or blood. Our previous research has shown that chloramine T, reacting in vitro with HSA, leads to the formation of AOPPs. Furthermore, this study also reported an increase in the level of AOPPs during pneumonia in COVID-19 patients [29]. Aggregation of plasma protein via disulfide bridges and/or dityrosine cross-linking in oxidative stress conditions can also affect mFLT. Taken together, it appears that oxidative stress may be responsible for at least part of the changes associated with the decrease in fluorescence lifetime observed in COVID-19 patients. However, more research is needed to confirm this hypothesis and fully understand the complex mechanisms underlying this phenomenon.

Another important modification of proteins that may alter mFLT is related to the creation of malondialdehyde (MDA)-protein adducts known as advanced lipoxidation end-products (ALEs). It has been revealed that higher lipid peroxidation and aldehydes levels are associated with increased severity of disease in COVID-19 patients [43,44]. MDA is a highly reactive compound that can readily react with amino groups of proteins such as HSA, leading to the formation of MDA-HSA adducts. However, this is just the first step in a series of reactions that can result in the formation of more complex ALEs with unknown molecular structures. Our earlier experiment showed that adding MDA to the HSA solution or plasma decreased the mFLT during incubation.

It is important to note that the interpretation of our results may be difficult due to variability in the relative protein composition of blood among individual patients. Human plasma contains a multitude of proteins, each with potentially different fluorescence lifetimes and emission quantum yields. The heterogeneous nature of plasma proteins and their varying concentrations can lead to significant differences in the contribution of each of them to the overall fluorescence signal of blood. The most abundant proteins, i.e., albumin and globulins, exert the greatest influence on the overall emission. During the acute phase of the systemic response to viral infection, inflammation, and tissue injury result in significant changes in the plasma protein content. Immunoglobulins, also known as gamma globulins, are involved in the immune response and protect against infections. Our measurements revealed an interesting finding that gamma globulins from human blood have a lower mFLT than HSA (2.7 vs. 5.3 ns). However, it should be noted that gamma globulins are not a homogeneous group of proteins and can vary depending on the severity of inflammation during SARS-CoV-2 infection. Different types of immunoglobulins can target specific viral proteins, including those found in SARS-CoV-2. Additionally, new variants of SARS-CoV-2 may require different types of immunoglobulins for effective neutralization. Since it is virtually impossible to create a perfectly controlled experiment in which the concentrations of all proteins in blood or plasma are equal, further research is required to investigate the fluorescence decay time of individual globulin fractions that play a critical role in the immune response to SARS-CoV-2 infection.

When analyzing the fluorescence of whole blood samples, it is important to consider the potential contributions of both red blood cells (RBCs) and white blood cells (WBCs). The autofluorescence of WBCs in the UV range is typically attributed to tryptophan primarily located in cellular organelles such as mitochondria and lysosomes. The fluorescence lifetime of individual WBCs subtypes is expected to differ due to variations in the intensity and shape of their autofluorescence spectra [45]. An increase in WBC count, neutrophilia, and a high neutrophil-to-lymphocyte ratio is commonly observed in severe COVID-19 cases [46,47]. However, there is limited information in the literature regarding the fluorescence lifetime of individual WBCs subtypes in the UV range. It is likely that the fluorescence lifetime of neutrophils changes after activation during oxidative bursts, which can occur in response to various stimuli, including viruses. Neutrophils are a type of white blood cell that play a critical role in the immune response to infections and are often the first cells to be recruited to sites of infection or tissue damage. The specific response of neutrophils to a virus can depend on various factors, such as the type of virus, the immune status of the individual, and the overall state of the immune system.

The autofluorescence of RBCs at a wavelength of 360 nm is primarily attributed to aromatic amino acids in the membrane proteins and, to a lesser extent, internal proteins. Changes in these proteins can potentially affect the fluorescence lifetime. Oxidative damage to RBC membrane proteins can impair their deformability, which is critical for their ability to deliver oxygen to tissues by squeezing through narrow capillaries in the body [48]. Several studies have reported protein damage, lipid remodeling, and changes in the size and rigidity of RBCs in COVID-19 patients [49,50]. HOCl can oxidize both the lipid structure and protein components of the erythrocyte membrane. Additionally, HOCl can lead to the formation of membrane pores and modify the permeability of ions across the membrane, further contributing to the hemolytic process. As a crucial antioxidant within the cell, glutathione (GSH) serves to neutralize oxidative damage. However, HOCl has the capability to convert GSH into its oxidized state, glutathione disulfide (GSSG), thereby weakening the cell’s defenses against subsequent oxidative assaults, which further intensifies the hemolysis [51,52,53]. Hemoglobin, which carries oxygen from the lungs to the body’s tissues, is another significant component in the blood. However, hemoglobin typically does not exhibit significant fluorescence in the ultraviolet (UV) region.

Finally, it is possible that infection by SARS-CoV2 could lead to the formation of new compounds or unknown fluorescent complexes, but there is currently no clear scientific evidence to support this hypothesis. This study has certain limitations that must be taken into account when interpreting the results. First, the blood samples were collected from only one hospital, which limits the generalizability of the findings. Second, critically ill patients requiring invasive mechanical ventilation were excluded from the study. To increase the validity of the findings, the sample size should be expanded in future studies to include patients with varying degrees of symptom severity. This would likely lead to stronger correlations between the mFLT and clinical parameters. Additionally, the sample size of patients examined in subsequent blood collections was reduced due to various factors, such as patient recovery or discontinuation of study participation. Although this technique has a lower cost per procedure and utilizes a less expensive methodology, the cost of the equipment required for this technique can be a limiting factor for its widespread use. Future research should focus on validating these findings in larger patient cohorts and exploring the potential of fluorescence lifetime measurements in monitoring treatment response and predicting patient outcomes. The integration of this technology into portable and user-friendly devices could enhance its utility in clinical practice, particularly in resource-limited settings and situations with a significant outbreak and a large number of infected patients.

## 4. Materials and Methods

### 4.1. General Characteristics of the Study Patients

For this study, we included 60 patients who were hospitalized with COVID-19 pneumonia in the Department of Lung Diseases, Neoplasms, and Tuberculosis at the Regional Center of Pulmonology in Bydgoszcz, Poland, between April and December 2021. The disease was confirmed by a positive reverse-transcription polymerase chain reaction (RT-PCR) test result from a nasopharyngeal swab according to the World Health Organization (WHO) criteria [54] and radiographic imaging. HRCTs were performed using a 64-slice Siemens Somatom Sensation (Siemens Healthcare, Erlangen, Germany) system with a slice thickness ≤ 0.5 mm, or chest X-ray. Patients with COVID-19 pneumonia and blood oxygen saturation levels below 94% on ambient air were eligible for inclusion in the study. However, those who were already receiving continuous positive airway pressure, bilevel positive airway pressure, or mechanical ventilation were excluded from the study. Basic laboratory tests assessing the advancement of inflammation, liver and kidney function, and coagulation system parameters were quantified using standard laboratory methods. The study group of COVID-19 patients were tested one week after their admission to the hospital, as long as they were in the acute phase of infection. This group consisted of 39 patients. The patients were asked to return for another round of blood sample collection after a six-month period, and a total of 27 individuals participated in this stage. Additionally, an examination was conducted on a group of 15 healthy individuals.

### 4.2. Sample Preparation

A blood sample was taken from each COVID-19 patient on the day of admission to the hospital department or the following day. All the blood samples were collected in standard sterile polystyrene tubes containing EDTA and processed within 2 h of collection. Initially, two aliquots of 5 µL volume were taken from each patient’s whole blood sample. Each aliquot was separately diluted 200 times in 995 µL phosphate-buffered saline (PBS) for measurements. The remaining blood was then centrifuged at 3500 rpm at 4 °C for 5 min to obtain plasma. The plasma fraction was collected and stored at −80 °C until measurement. To preserve sample integrity, multiple freeze–thaw cycles were avoided. All plasma measurements were performed on the same day within 1 h of defrosting the sample. For the measurement process, the plasma was diluted 20 times in PBS (50 µL in 950 µL of PBS).

### 4.3. AOPPs Measurements

According to the modified method described by Witko-Sarsat [55], the AOPP level was determined by measuring the absorbance at 340 nm. To perform the AOPP assay, a reactant mixture containing 1.875 mL of 0.2 M citric acid and 25 µL of 1.16 M potassium iodide was prepared. Subsequently, 1.9 mL of the mixture was combined with 100 µL of plasma, and the absorbance was immediately recorded. The use of citric acid instead of acetic acid improved the stability of the modified method over time [56]. The results were expressed as chloramine T equivalents.

### 4.4. In Vitro Study

All ingredients were purchased from Merck Life Science Sp.z.o.o (Poznań, Poland). A 10 µM concentration of human gamma globulin and human serum albumin (HSA) suspended in PBS at pH 7.4 were used for the fluorescence lifetime measurements. Oxidation of protein was created in vitro by treating purified HSA (10 µM) with chloramine T at different concentrations for 60 min.

### 4.5. Time-Resolved Fluorescence Spectroscopy Measurements

A time-resolved spectrofluorometer, Life Spec II (Edinburgh Instruments Ltd., Livingston, UK), with a sub-nanosecond pulsed EPLED^®^ diode emitting light of 280-nanometre wavelength was used to measure the fluorescence lifetime of blood and plasma. For the plasma or HSA and blood samples, the exposure time was 1 and 5 min, respectively. Fluorescence measurements of the samples were taken at 360 nm after bringing them to room temperature before performing the assay. The measurements of 1 mL samples were carried out using quartz 3.5 × 10 mm cuvettes. The fluorescence decay of the blood samples was recorded for two different blood donations from the same patient. For the plasma samples, only one measurement was performed per patient. The fluorescence lifetime of HSA and its oxidized forms in the presence of chlorine compounds was measured once. The fluorescence lifetimes were obtained by deconvolution analysis of the data using the multiexponential model of the fluorescence decay, and the instrument response function was taken into account. Then, the mFLT was calculated as the weighted average of fluorescence lifetimes obtained from the three- and four-exponential models of fluorescence decay for plasma or HSA and blood, respectively. By averaging weights, the contributions of individual components (areas under decay curves) to the total fluorescence were obtained. The appropriate number of exponents was determined based on chi-square (χ^2^) statistical analysis and the visual assessment of residual plots.

### 4.6. Statistical Analysis

Data analysis was carried out to identify potential differences or correlations between the investigated parameters. Firstly, the normality of these parameters was tested using the Shapiro–Wilk test. Since some of the variables were found to be non-normal, non-parametric tests were used. Spearman’s rank correlation coefficients (r values) were applied to determine dependencies between the fluorescence lifetime parameters and clinical biomarkers. The Wilcoxon signed-rank test was used to find significant differences between mFLT measurements taken at different times (on admission, one week later and 6 months post-infection) in the COVID-19 group. For independent samples, the Mann–Whitney U test was applied. Differences and correlations were considered significant if the *p*-value was less than 0.05.

## 5. Conclusions

The method presented in this study offers a direct, repeatable, non-invasive, and time-efficient technique, requiring only simple sample pre-treatment without additional reagents. Its low volume requirement also enables the use of fingertip blood samples through capillary sampling. In vitro, studies involving HSA have shown that a decrease in the fluorescence lifetime of blood and plasma could be related to albumin oxidation during the acute phase of inflammation. The observed correlations between blood or plasma mFLT levels and inflammatory parameters or surrogate markers of COVID-19 severity, such as albumin, as well as lung HRCT scores and AOPPs, confirm that time-resolved fluorescence spectroscopy is a promising tool for detecting and assessing the severity of SARS-CoV-2 pneumonia. Moreover, the measurement of mFLT could have significant clinical value in predicting sepsis, a condition typically associated with higher levels of oxidative stress. Thus, conducting the test as early as possible is advisable. To the best of our knowledge, this is the only study to have examined the fluorescence lifetime of whole diluted blood in cases of acute infection. While our method shows promise in identifying inflammation in COVID-19, its utility as an early detection biomarker for COVID-19 infection requires further rigorous evaluation.

## Figures and Tables

**Table 1:** Levels of significance (*p*-values) of difference between the study groups. C, COVID-19 patients on admission to hospital; C-1W, COVID-19 patients 1 week upon admission; C-6M, COVID-19 patients 6 months post-infection; H, Healthy. Red-colored values indicate statistical significance (*p* < 0.05).

		C	C-1W	C-6M	H
blood	C	-	<0.001	<0.001	<0.001
	C-1W	<0.001	-	0.003	<0.001
	C-6M	<0.001	0.003	-	0.478
plasma	C	-	0.199	<0.001	<0.001
	C-1W	0.199	-	0.001	<0.001
	C-6M	<0.001	0.001	-	0.937

**Table 2:** Demographic, clinical, and laboratory parameters and their correlations with mFLT of blood and plasma of studied patients with COVID-19 pneumonia on admission. Red-colored values indicate statistical significance (*p* < 0.05). mFLT, mean fluorescence lifetime; WBC, White blood cells; RBC, Red blood cells; Hgb, Hemoglobin; PLT, Platelets count; CRP, C-reactive protein; LDH, Lactate dehydrogenase; CPK, Creatinine phosphokinase; AST, Aspartate transaminase; ALT, Alanine transaminase; IL-6, Interleukin-6; HRCT, High-resolution computed tomography; AOPPs, Advanced oxidation protein products; AGR, albumin to globulin ratio.

Parameters (Units)	Median	Interquartile Range	Reference Ranges	Correlation with mFLT (r)	
				Blood	Plasma
Age (years)	65	52–72		−0.173	−0.229
Symptoms (days)	7	5–10		0.024	0.062
WBC (10^3^/µL)	6.25	5.05–9.1	4–10	−0.266	−0.183
Neutrophils (10^3^/µL)	4.6	3.5–7.3	2.5–5	−0.195	−0.097
Lymphocytes (10^3^/µL)	0.9	0.7–1.3	1.5–3.5	−0.159	−0.042
RBC (10^6^/µL)	4.5	4.2–4.8	4.5–5.5	0.249	0.294
Hgb (g/dL)	13.8	12.8–14.5	14–18	0.136	0.175
PLT (10^3^/µL)	204.5	168.5–290.5	130–350	−0.212	−0.185
CRP (mg/L)	81.5	41–138	<5	−0.383	−0.216
Procalcitonin (ng/mL)	0.08	0.05–0.15	<0.05	−0.417	−0.208
LDH (U/L)	639.5	525.5–778	225–450	−0.405	−0.143
D-Dimers (ng/mL)	900	708–1546	<500	−0.524	−0.464
Troponin (ng/L)	10.1	6–18	<19	−0.401	−0.316
Creatinine (mg/dL)	0.955	0.855–1.11	0.8–1.3	0.067	0.017
CPK (U/L)	128	75.5–209	25–200	0.180	0.099
AST (U/L)	50.5	35.5–65	<37	−0.390	−0.306
ALT (U/L)	42	30–61	<40	−0.192	−0.079
IL-6 (pg/mL)	13.1	4.9–35	<7	−0.023	0.016
HRCT score	0.25	0.15–0.42		−0.340	−0.199
Albumin (g/L)	34	31–37	39–51	0.407	0.434
AOPPs (µM)	13.7	11.4–16.4		−0.361	−0.268
AGR	1.25	1.07–1.40		0.355	0.588

## Data Availability

The data presented in this study are available on request from the corresponding author.
